# Targeting oncogenic PLCE1 by miR-145 impairs tumor proliferation and metastasis of esophageal squamous cell carcinoma

**DOI:** 10.18632/oncotarget.6499

**Published:** 2015-12-08

**Authors:** Xiao-Bin Cui, Su Li, Ting-Ting Li, Hao Peng, Ting-Ting Jin, Shu-Mao Zhang, Chun-Xia Liu, Lan Yang, Yao-Yuan Shen, Shu-Gang Li, Na Li, Yong Li, Jian-Ming Hu, Jin-Fang Jiang, Jing Suo, Yan Qi, Wei-Hua Liang, Liang-Hai Wang, Hong-Wei Dang, Li Li, Wei-Wei Cao, Yutao Wei, Laibo Yin, Chuan-Yue Wu, Xiang-Lin Yuan, Hong Zhou, Yu Zheng, Yun-Zhao Chen, Feng Li

**Affiliations:** ^1^ Department of Pathology and Key Laboratory for Xinjiang Endemic and Ethnic Diseases, Shihezi University School of Medicine, Shihezi, China; ^2^ Department of Oncology, Tongji Hospital, Huazhong University of Science and Technology, Wuhan, China; ^3^ Department of Pathology, Fenyang College, Shanxi Medical University, Fenyang, China; ^4^ Department of Pathology, People Hospital of Xinjiang Uygur Autonomous Region, Urumqi, China; ^5^ Department of Oncology, The First Affiliated Hospital, Shihezi University School of Medicine, Shihezi, China; ^6^ Department of CT and MRI, The First Affiliated Hospital, Shihezi University School of Medicine, Shihezi, China; ^7^ The Key Laboratory of Xinjiang Endemic and Ethnic Diseases, Shihezi University School of Medicine, Shihezi, China; ^8^ Department of Thoracic and Cardiovascular Surgery, The First Affiliated Hospital, Shihezi University School of Medicine, Shihezi, China; ^9^ Department of Pathology, University of Pittsburgh, Pittsburgh, PA, USA; ^10^ Bone Research Program, ANZAC Research Institute, University of Sydney, New South Wales, Australia

**Keywords:** PLCE1, miR-145, esophageal carcinoma, proliferation, invasion

## Abstract

Phospholipase C epsilon 1 (PLCE1) is a susceptibility gene in esophageal squamous cell carcinoma (ESCC). Nevertheless, the role of PLCE1 in ESCC tumorigenesis has not been elucidated. In this study, we determined the function of PLCE1 and its regulatory microRNA (miRNA) in ESCC. PLCE1 protein was excessively expressed in ESCC and precancerous lesions compared with that in normal tissues. High PLCE1 expression levels in ESCC were significantly linked with poor overall survival. Knockdown of PLCE1 promoted the apoptosis, cytokine-induced apoptosis, and sensitivity of cancer cells to chemotherapeutic drugs but abrogated the proliferation and EMT phenotype of ESCC *in vitro*. Notably, miR-145 was newly identified as a potent repressor of PLCE1 expression by directly targeting the 3′UTR of PLCE1. MiR-145 also inhibited cell proliferation, migration, and metastasis, as well as controlled the cytoskeleton dynamics of esophageal cancer. Moreover, miR-145 was expressed at low levels in a large cohort of patients with ESCC and was inversely correlated with PLCE1 protein expression in cancer cells and tissues. These findings demonstrate that PLCE1 functions as tumor promoter in ESCC and can be suppressed by miR-145 through inhibition of PLCE1 translation. Hence, delivery of PLCE1-targeting miR-145 is a potential therapeutic approach for esophageal cancer.

## INTRODUCTION

Esophageal squamous cell carcinoma (ESCC), a tumor of the digestive tract, is an aggressive malignancy with poor patient survival in China. Esophageal cancer is the eighth most common cancer and the sixth most common causes of cancer mortality worldwide [1]. Patients with ESCC exhibit poor survival, with an overall 5-year survival rate following surgery of only 14% to 22%. Therapeutic strategies for this cancer type remain limited because of poor understanding of ESCC pathogenesis. Invasion and metastasis of cancer cells remain prevalent in ESCC, despite advances in ESCC treatments, particularly in terms of surgery, chemotherapy, radiation, or combination of these options [2, 3]. Studies demonstrated that several oncogenic and tumor-suppressive factors are associated with ESCC progression. However, only few of these studies are specific and conclusive, and the molecular pathogenesis of ESCC remains poorly understood [4, 5]. Therefore, the biological behavior of the initiation and progression of esophageal cancer, especially ESCC, must be elucidated to develop effective diagnostic methods and therapeutic strategies.

The three-scale genome-wide association studies of Chinese Han populations identified a new susceptibility locus related to ESCC in the phospholipase C epsilon 1 (PLCE1), a member of the phospholipase family [6–9]. This locus contains several Ras interactive domains, including one CDC25 domain and two Ras-associating domains [10], and is located in chromosome10q23, which is a frequently amplified region in cancer. The structure of PLCE1 renders it with functions related to genome regulation, including cell growth, differentiation, apoptosis, and angiogenesis [11, 12]. In our previous study, we found that the genetic variants of PLCE1 are candidate makers for ESCC susceptibility of the Kazakh population. And these linkage disequilibrium variants may influence ESCC risk individually and jointly by promoting the mRNA and protein expression levels of PLCE1 [9]. We also confirmed that the heterozygote of PLCE1 rs2274223 increase susceptibility to HPV infection in Kazakh patients with esophageal carcinoma [13]. Studies also revealed that PLCE1 plays crucial roles in several tumor types, such as gastric [14–16], bladder [17], head and neck [18], gallbladder [19], and colorectal cancer [20, 21]. However, research on PLCE1 expression in ESCC provides contradicting findings. A study showed that PLCE1 protein expression level is higher in ESCC tissues than that in normal tissues in Chinese Han ethnic group [7]. In our previous study, we confirmed that PLCE1 plays a tumor-oncogenic function in ESCC and is frequently upregulated in Kazakh patients with ESCC [22]. By contrast, Hu *et al.* [23] showed that PLCE1 mRNA expression level is lower in ESCC than that in normal tissues. Moreover, the IHC score of ESCC is not significantly different from that of the normal match. Therefore, further research must be performed to understand the precise role of the susceptibility gene PLCE1 in ESCC. The underlying mechanism of PLCE1dysregulation in ESCC must also be investigated.

MicroRNAs (miRNAs), a class of small non-coding RNAs with 20 to 22 nucleotides, regulate gene expression at the post-transcriptional level by binding to the 3′-untranslated region (UTR) of the target mRNAs, leading to mRNA degradation or translation inhibition [24, 25]. MiRNA are aberrantly expressed in various cancers and function as a novel class of oncogenes or tumor suppressor genes depending on their targets [26]. In ESCC, the aberrant expressions level of miRNAs, such as miR-27a, miR-9, miR-335, and miR-183, regulate tumor cell growth, apoptosis, migration, and invasion by targeting proteins involved in these cellular pathways [27–30]. Thus far, miRNAs that selectively regulate PLCE1 in ESCC have not been identified. In this study, we reported that high PLCE1 expression levels in ESCC are significantly correlated with poor patient survival. Overexpressing PLCE1 potently stimulates cancer cell growth and invasion and promotes esophageal tumorigenesis in ESCC. We also identified for the first time that PLCE1 is a potential target of miR-145, whose expression was aberrantly downregulated in patients with ESCC from the Han and Kazakh ethnic groups and inversely correlated with PLCE1 expression. Notably, enhancing miR-145 expression could impair tumor proliferation and metastasis of esophageal cancer. Thus, the present mechanistic study indicates that delivery of PLCE1-targeting miR-145 is a candidate therapeutic approach for preventing tumor proliferation and metastasis of esophageal cancer.

## RESULTS

### Enhanced PLCE1 expression is correlated with ESCC aggressiveness and poor patient survival

**Figure 1:**
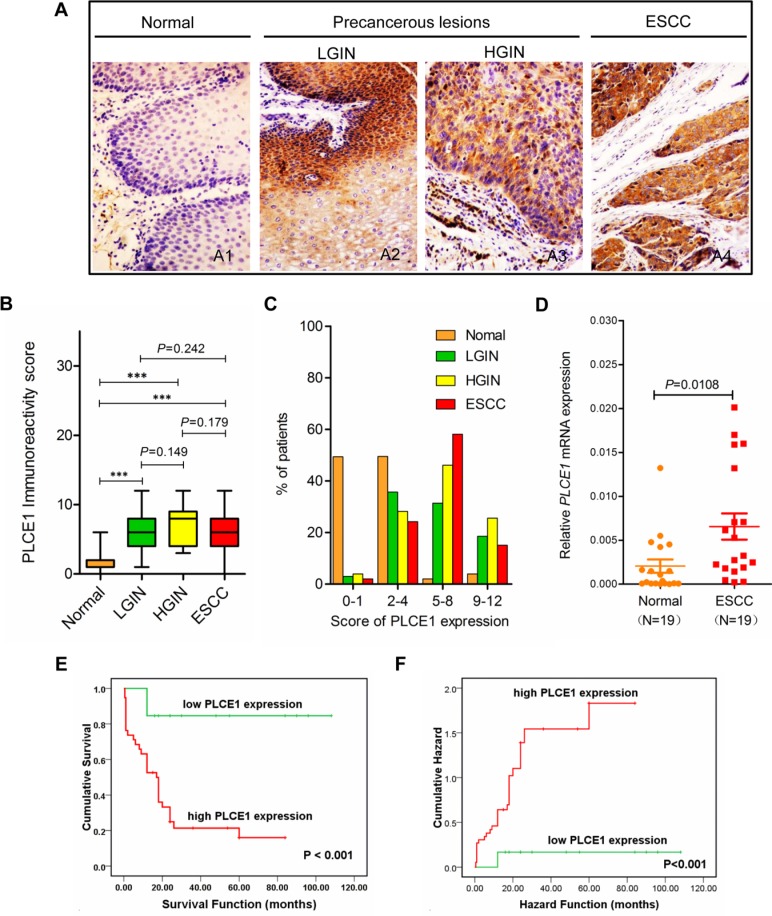
Increased PLCE1 protein expression is linked with ESCC aggressiveness and poor patient survival Representative PLCE1 immunostaining in (**A1**) morphologically normal operative margin tissues; precancerous lesions: (**A2**) LGIN and (**A3**) HGIN; and (**A4**) ESCC tissues. Original magnification for A1–A4, x200. (**B**) Boxplot analysis using Student's *t*-test of PLCE1 immunoreactivity scores in normal human esophageal squamous epithelium, precancerous lesions, and ESCC tissues. The box refers to the quartile distribution (25%–75%) range, with the median represented by a black horizontal line. *P < 0.05; **P < 0.01; ***P < 0.001. (**C**) Distribution of four-level scores (0–1, 2–4, 5–8, and 9–12) of PLCE1 protein expression in normal, precancerous lesions, and ESCC. (**D**) Real-time PCR analysis of PLCE1 mRNA expression in 19 pairs of fresh ESCC tissues and adjacent morphologically normal operative margin tissues. (**E**) Log-rank test revealed that patients in the low-expression PLCE1 group exhibited significantly higher survival rate than those in the high-expression group (*P* < 0.001). (**F**) Patients with ESCC showing PLCE1 overexpression are at a higher risk of death than those showing lower PLCE1 levels (*P* < 0.001).

Our previous study reported an increased PLCE1 expression in Kazakh patients with ESCC [31]. Nevertheless, the presence of PLCE1 expression in precancerous lesions and its prognostic significance in ESCC have not been examined. Therefore, in the present study, we investigated PLCE1 expression in precancerous lesions and assessed its correlation with survival of patients with ESCC. Figure 1 shows that most esophageal tumors and precancerous lesions exhibited strong cytoplasmic staining for PLCE1, whereas only few cells of normal esophageal tissues showed positive staining for PLCE1 (Figure 1A). The patients were then dichotomized into two categories according to their immunoreactivity for PLCE1. PLCE1 protein was upregulated in 73.22% (82/112) of ESCC, 72.50% (28/40) of HGIN, 58.33% (35/60) of LGIN, and 2.03% (2/99) of normal epithelium, thereby indicating gradual increase in PLCE1 expression from the normal esophageal epithelium to ESCC (Supplementary Table 1, Figure 1B). The distribution of four-level scores (0–1, 2–4, 5–8, and 9–12) of PLCE1 protein expression significantly differed between normal precancerous lesions and ESCC (Figure 1C). We also investigated the mRNA expression of PLCE1 by using 19 pairs of fresh ESCC tissues and their corresponding morphologically normal tissues through qRT-PCR. The results showed that the mean mRNA level of PLCE1 was threefold higher in ESCC samples than that in the corresponding normal esophageal epithelial tissues (0.006556 ± 0.0015 vs. 0.002051 ± 0.0007, *P* = 0.0108, Figure 1D). Kaplan–Meier survival analysis also revealed that the overall survival rate was significantly lower in patients with high PLCE1 expression than that in patients with low PLCE1 expression (log-rank test, χ2 = 6.749, *P* < 0.001, Figure 1E and 1F). Moreover, multivariate survival analysis using Cox's proportional hazards model showed a close correlation between high PLCE1 protein expression and clinical prognosis (HR = 8.435, 95% CI = 1.875 to 37.983, *P* = 0.005, Supplementary Table 2). These findings indicate that PLCE1 overexpression is a poor prognostic marker in patients with ESCC.

### PLCE1 downregulation suppresses ESCC cell growth and induces apoptosis

**Figure 2:**
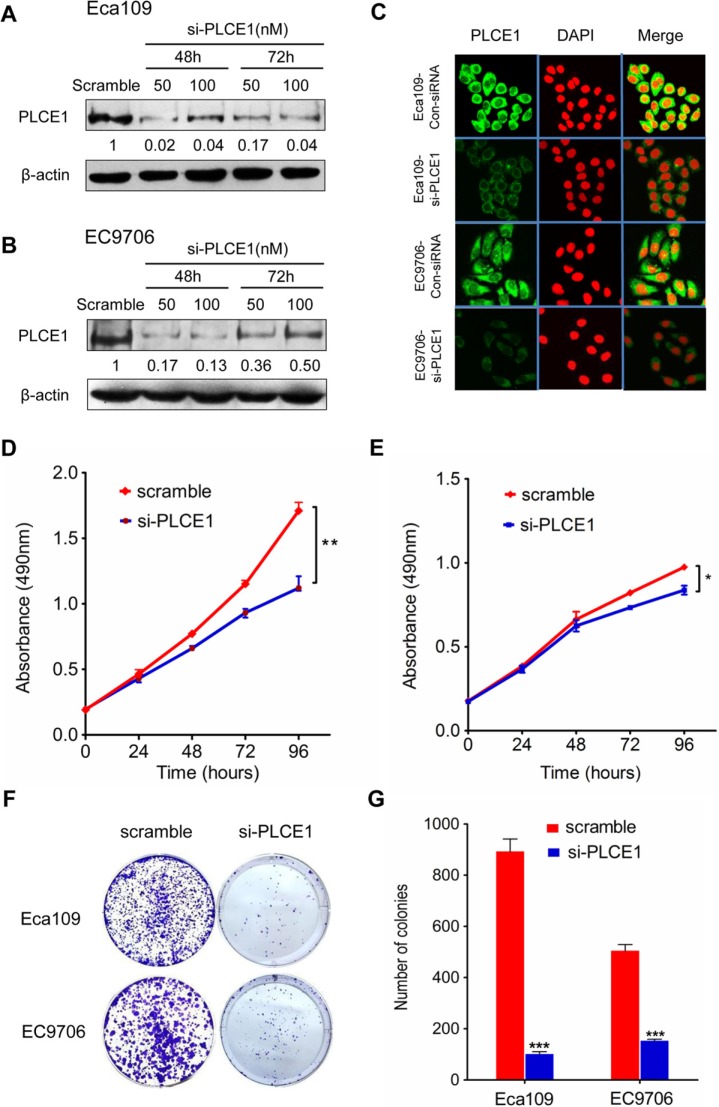
PLCE1 inhibition reduces cell proliferation and colony formation potential in ESCC cell lines (**A**, **B**, and **C**) Suppression of PLCE1 protein by siRNA transfection was confirmed by Western blot analysis and immunofluorescent staining in ESCC cell lines Eca109 and EC9706. β-actin was used as the loading control. (**D** and **E**) Proliferation ability of si-PLCE1-transfected ESCC cell lines: (D) Eca109 and (E) EC9706, as revealed by MTT assay. *P < 0.05; **P < 0.01 vs. scramble control (Student's *t*-test). (**F**) Effects of si-PLCE1 on colony formation of Eca109 and EC9706 ESCC cells. (**G**) Statistics of the colony number in (F). ***P < 0.001 vs. scramble control (Student's *t*-test).

**Figure 3:**
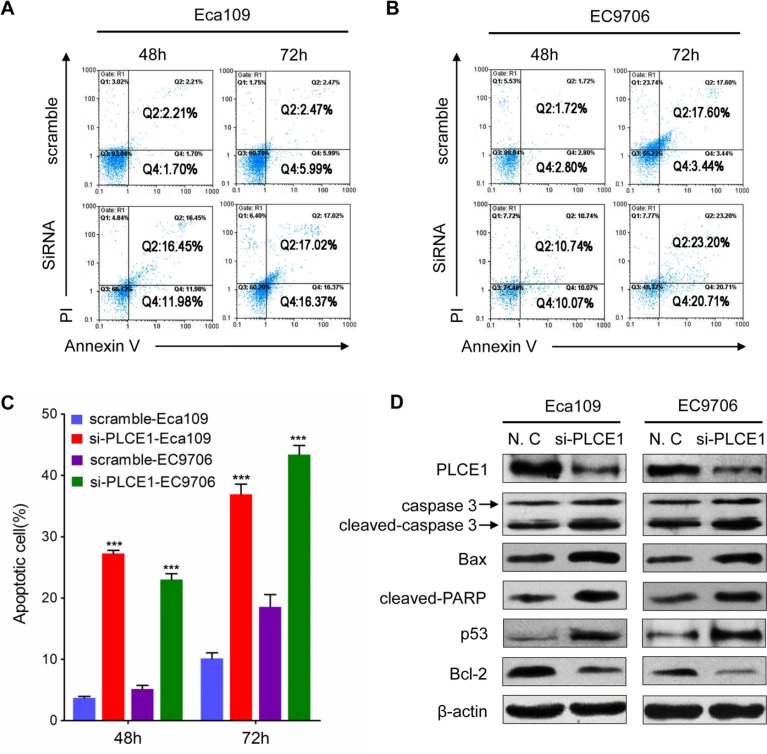
PLCE1 knockdown enhances ESCC apoptosis (**A** and **B**) FACS analysis of apoptotic Eca109 and EC9706 cells upon PLCE1 inhibition. (**C**) Statistics of results in A and B. ***P < 0.001 vs. scramble control (Student's *t*-test). (**D**) Western blot analysis was used to compare the expression levels of apoptosis-related proteins in Eca109 and EC9706 cells with knocked down PLCE1 with those of the negative control.

To investigate the biological functions of PLCE1 in the proliferation and transformation of malignant esophageal squamous cells, we knocked down PLCE1 through RNA interference. We also conducted Western blot and flow cytometry analyses, as well as MTT and colony formation assays, to determine protein expression, apoptosis, growth rate, and proliferation rate, respectively, of ESCC cell lines Eca109 and EC9706. The PLCE1 protein levels were successfully reduced through transfection of specific siRNA against PLCE1 (Figure 2A–2C). The proliferation rate of Eca109 and EC9706 cells decreased in si-PLCE1-transfected cells compared with that in the respective controls (Figure 2D and 2E). Similarly, colony formation assays showed that the number of colonies decreased following PLCE1 downregulation in the two ESCC cell lines (Figure 2F and 2G). In addition, flow cytometry analysis results showed that early apoptotic rate increased by 8.3- and 4.1-fold in Eca109-si-PLCE1 and EC9706-si-PLCE1 cells, respectively (Figure 3A–3C). Furthermore, we determined changes in the expression of apoptosis-related proteins following PLCE1 silencing in ESCC cells. Western blot analysis showed that PLCE1 inhibition increased the levels of the apoptosis-related protein p53, Bax, cleaved PARP, caspase3, and cleaved caspase3 but decreased the level of Bcl-2 (Figure 3D). This finding indicates that PLCE1 may play a critical role in anti-apoptosis by negatively regulating the level of the p53-mediated pro-apoptotic signaling pathway.

### PLCE1 induces ESCC cell migration and invasion and controls cytoskeleton dynamics

**Figure 4:**
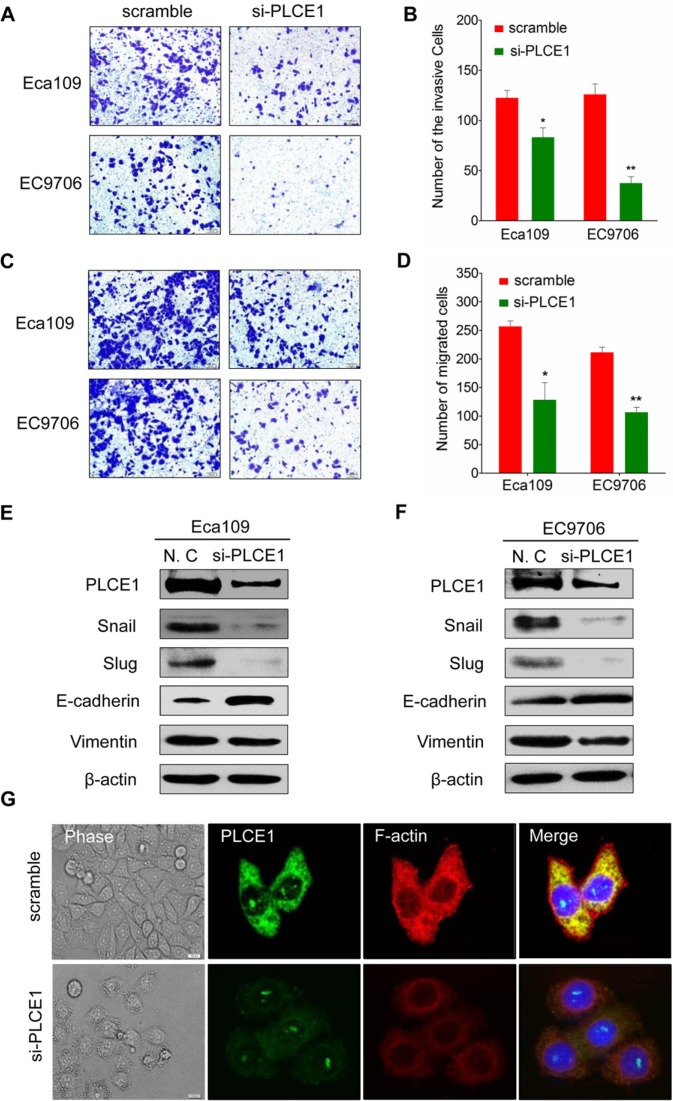
PLCE1 inhibition reduces ESCC migration and invasion and regulates ESCC cytoskeleton dynamics (**A** and **B**) PLCE1 depletion considerably inhibited cell invasion and migration in both cell lines. (**C** and **D**) The numbers of invading and migrating cells were counted, and a significant difference was observed between the cell lines. *P < 0.05; **P < 0.01. (**E** and **F**) EMT-related markers showed different expression levels in Eca109 and EC9706 cells transfected with and without si-PLCE1. (**G**) PLCE1 knockdown inhibited lamellipodia and filopodia formation and ESCC cell distribution. The morphology of Eca109 treated with si-PLCE1 was analyzed by phase contrast microscopy (phase). PLCE1 (green) and F-actin (red) distribution was analyzed in fixed cells by immunofluorescence microscop.

The tumor cell migration and invasion assay indicated that si-PLCE1 transfection reduced the invasion and migration capability of Eca109 and EC9706 cell lines. The number of invading and migrating cells was significantly lower in si-PLCE1-treated cells than that in the nonsense-siRNA groups (Figure 4A–4D). Individual cell migration and invasion of surrounding tissues are promoted by epithelial–mesenchymal transition (EMT), a condition where cells lose their epithelial characteristics to acquire the appearance and behavior of mesenchymal cells. The expression of EMT markers, namely, the epithelial cell marker E-cadherin and the mesenchymal marker vimentin, in si-PLCE1-transfected Eca109 and EC9706 cells and in the controls were examined using Western blot analysis to investigate whether PLCE1 promotes EMT in esophageal cancer cells. After knocked down of PLCE1, E-cadherin expression was significantly upregulated, whereas vimentin expression was significantly downregulated in the two cell lines compared with that in the control siRNA groups (Figure 4E and 4F). Our data also showed that PLCE1 inhibition reduced the expression of EMT-inducing transcription factors, namely, Slug and Snail (Figure 4E and 4F). Moreover, cell migration and invasion in cancer is influenced by cell motility. Examination of cell morphology indicated that PLCE1 knockdown induced morphological changes in Eca109 cells (Figure 4G). Furthermore, the use of phalloidin-TRITC (F-actin) to monitor cytoskeleton dynamics in Eca109 cells showed that PLCE1 knockdown inhibited the formation of lamellipodia and filopodia compared with that in control cells (Figure 4G). These results indicate that PLCE1 may stimulate esophageal cancer cell motility and migration by promoting EMT and controlling cytoskeleton dynamics.

### PLCE1 contributes to ESCC cell resistance to apoptosis induced by TNFα, TRAIL, paclitaxel, and fluorouracil (5-FU)

**Figure 5:**
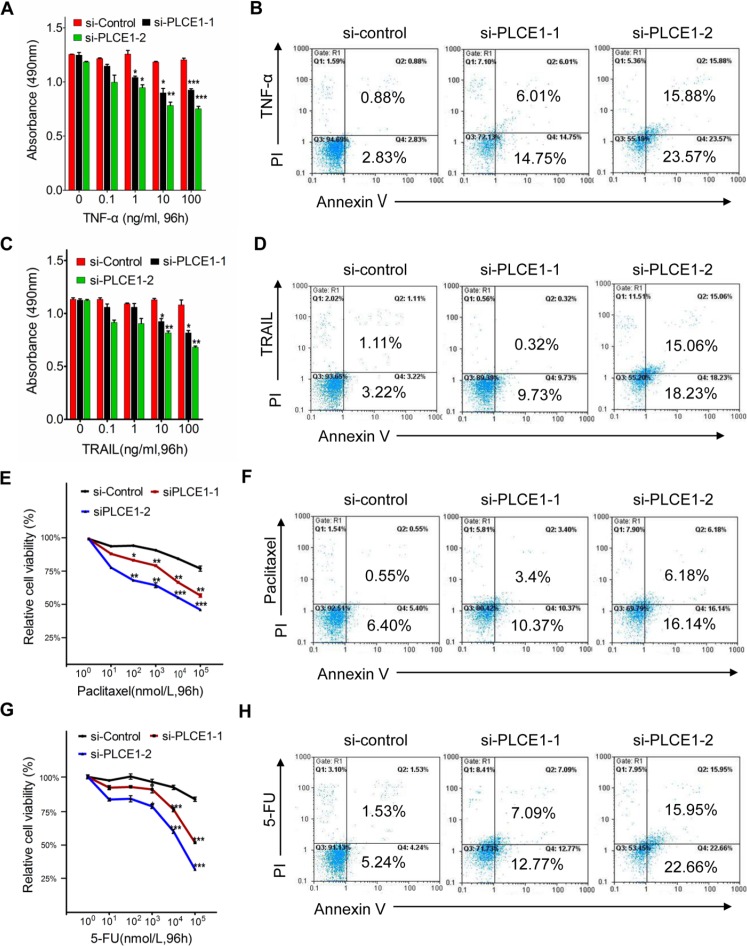
PLCE1 knockdown enhances apoptosis induced by TNFα, TRAIL, paclitaxel, and 5-FU (**A**, **C**, **E** and **G**) Quantification of cell numbers (by MTT) in cell cultures treated with indicated concentrations of TNF-α (A), TRAIL (C), paclitaxel (F), and 5-FU (G). *P < 0.05; **P < 0.01; ***P < 0.001 vs. scramble control (Student's *t*-test). (**B**, **D**, **F**, and **H**) FCM analysis of apoptotic cells with Annexin V-FITC and propidium iodide staining 48 h after the respective treatments.

Our previous research demonstrated that upregulation of PLCE1 is correlated with increased expression of NF-κB related proteins in Kazakh patients with ESCC [32], Our previous research demonstrated that PLCE1 upregulation is correlated with enhanced expression of NF-κB-related proteins in Kazakh patients with ESCC; hence, the interaction between PLCE1 and NF-κB signal pathway may be involved in ESCC carcinogenesis. Increased NF-κB levels, which are frequently observed in human cancers, including esophageal cancer, may improve tumor cell survival. As such, we investigated the effect of PLCE1 knockdown in ESCC cells on TNFα-mediated NF-κB activation and apoptotic induction. The results showed that TNF-α dose-dependently decreased cell viability and increased cell apoptosis rate in si-PLCE1-transfected Eca109 cells compared with those in si-control Eca109 cells (Figure 5A and 5B). We then compared the responses of si-PLCE1-transfected Eca109 cells and si-control Eca109 cells to TRAIL-induced apoptosis. TRAIL strongly induced the apoptosis of si-PLCE1-transfected Eca109 cells compare with that of si-control cells (Figure 5C and 5D). Response of ESCC cells to apoptosis induced by cytokines following PLCE1 knockdown indicated that PLCE1 inhibition may sensitize the cells to apoptosis induced by chemotherapeutic agents.

We also determined the effect of PLCE1 knockdown on the survival of cancer cells given with Paclitaxel and 5-FU, which are chemotherapeutic drugs commonly used for clinical treatment of ESCC. PLCE1 knockdown induced Eca109 cells to be sensitive to paclitaxel-induced cell death (Figure 5E and 5F). Similarly, suppression of PLCE1 protein expression in Eca109 rendered the cells to be sensitive to 5-FU-induced cell death and apoptosis (Figure 5G and 5H). These findings indicate that PLCE1 knockdown promoted apoptosis induced by TNFα, TRAIL, paclitaxel, and 5-FU; hence, overexpressing PLCE1 contributed to the resistance of ESCC cells to chemotherapy.

### PLCE1 is a direct target of miR-145 in ESCC

**Figure 6:**
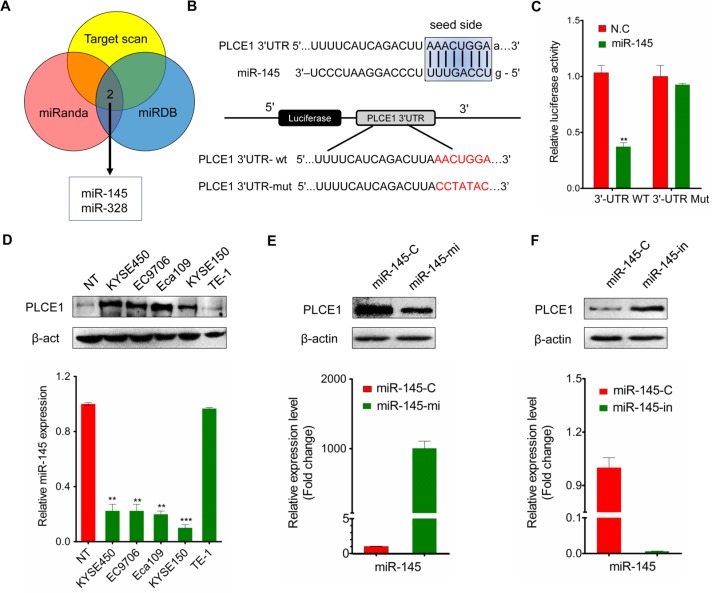
MiR-145 targets PLCE1 in ESCC (**A**) Online miRNA target prediction databases (TargetScan, miRanda, and miRDB) were used to predict miRNAs targeting PLCE1. (**B**) RNAhybrid prediction of the binding sites of miR-145 in the 3′-UTR of PLCE1. (**C**) Luciferase reporter assay. Wild-type (3′-UTR WT) and mutant PLCE1 3′-UTR (3′-UTR Mut) constructs were inserted into the pMIR-PLCE1 luciferase plasmid, and miR-145 was transfected into ESCC cells. (**D**) Inverse correlation between PLCE1 protein expression and miR-145 expression in ESCC lines. Upper panel: PLCE1 protein expression assessed by Western blot analysis. Lower panel: relative miR-145 expression level in ESCC lines measured by qPCR. (**E**) PLCE1 protein expression and miR-145 expression measured by qPCR 48 h after transfection of miR-145 in Eca109. Lower panel: the relative miR-145 expression level was upregulated in miR-145-transfected Eca109 cells. Upper panel: PLCE1 protein was downregulated in miR-145-transfected Eca109 cells. (**F**) PLCE1 protein expression and miR-145 expression measured by qPCR 48 h after transfection of miR-145–in in TE-1. Lower panel: the relative miR-145 expression level was downregulated in miR-145-in-transfected TE-1 cells. Upper panel: PLCE1 protein was upregulated in miR-145-in-transfected TE-1 cells.

Enhanced PLCE1 expression contributed to aggressive esophageal cancer growth and migration. Molecular pathways leading to PLCE1 overexpression are potentially important oncogenic mechanisms for esophageal cancer development. To determine whether miRNAs upregulate PLCE1 expression in esophageal cancer, we determined miRNAs targeting PLCE1 by using online miRNA target prediction databases (TargetScan, miRanda, and miRDB). These databases predicted that miR-145 potentially targets PLCE1 (Figure 6A). We then performed a bio-informatic analysis using RNAhybrid and TargetScan to determine whether PLCE1 is a direct target of miR-145. The analysis showed a complementary match between the miR-145 seed sequence and the 3′-UTR of PLCE1 (Figure 6B). Dual-luciferase reporter assays also showed that miR-145 significantly attenuated the luciferase activity of the reporter vector containing the wt-3′-UTR of PLCE1, whereas this effect was abrogated when the 3′-UTR binding site was mutated (Figure 6C). These findings indicate that miR-145 directly suppressed PLCE1 expression by binding to the 3′-UTR of PLCE1.

Given that PLCE1 was significantly upregulated in ESCC tissues, we hypothesized that the ectopic expression of miR-145 can induce PLCE1 inactivation in ESCC. As such, we investigated the endogenous expression of miR-145 and PLCE1 protein in various ESCC cell lines. Most ESCC cancer cell lines exhibited significantly low endogenous miR-145 expression levels compared with normal tissues; by contrast, other esophageal cancer cell lines, such as TE-1, expressed relatively high endogenous miR-145 expression levels similar to normal tissues (Figure 6D, lower panel). The endogenous PLCE1 protein level in TE-1 was then determined to clarify whether endogenous miR-145 expression replicates PLCE1 protein expression. Similar to that in normal tissues, PLCE1 expression was nearly non-detectable in TE-1 cells but highly upregulated in Eca109, EC9706, KYSE450, and KYSE150 cell lines (Figure 6D, upper panel).

Basing on the significant negative correlation between endogenous miR-145 and PLCE1 expression in ESCC cell lines, we hypothesized that ectopic miR-145 expression also significantly affects PLCE1 protein expression. miR-145 expression levels increased in Eca109 cells transfected with miR-145 mimic (Figure 6E, lower panel) but decreased in TE-1 cells transfected miR-145 inhibitor (Figure 6F, lower panel). Meanwhile, Western blot analysis revealed that PLCE1 protein expression decreased in miR-145 mimic-transfected Eca109 cells (Figure 6E, upper panel) but increased after miR-145 inhibition in TE-1 cells (Figure 6F, upper panel). These findings further demonstrate the potential significance of miR-145 as a determinant of PLCE1 expression in esophageal cancer cells.

### MiR-145 functions as tumor suppressor by directly inhibiting oncogenic PLCE1 in ESCC

**Figure 7:**
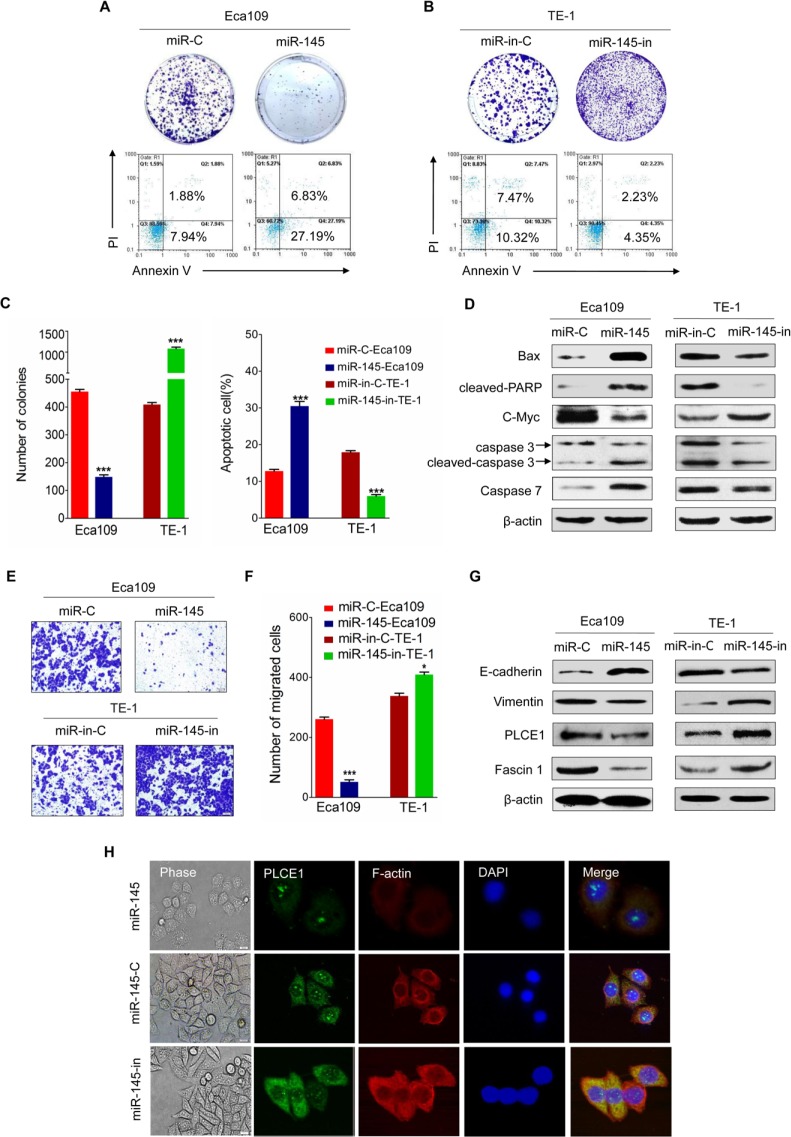
Tumor suppressive effects of miR-145 in ESCC (**A**) Representative results of colony formation assay and FACS analysis of apoptotic miR-145 mimic-treated Eca109 cell. (**B**) Representative results of colony formation assay and FACS analysis of apoptotic miR-145 inhibitor-treated TE-1 cells. (**C**) Statistics of the results in A and B. *P< 0.05, **P < 0.01, ***P < 0.001 vs. scramble control (Student's *t*-test). (**D**) Expression of apoptosis-related proteins such as Bax, cleaved PARP, caspase3, cleaved caspase3, and c-Myc protein, as measured by Western blot analysis in the indicated ESCC cell lines. (**E**) Effects of miR-145 on migration of Eca109 and TE-1 ESCC cells. (**F**) Statistics of the results in E. *P < 0.05; **P < 0.01 vs. scramble control (Student's *t*-test). (**G**) EMT-related markers and Fascin1 show different expression levels in Eca109 and TE-1 cells with and without miR-145 overexpression. (**H**) The morphology of Eca109 treated with miR-145 mimic or miR-145 inhibitor was analyzed by phase contrast microscopy (phase). PLCE1 (green) and F-actin (red) distribution was analyzed in fixed cells by immunofluorescence microscopy.

The ESCC cell lines were transfected with miR-145 mimic or miR-145 inhibitor to elucidate the antitumor effects of miR-145 in ESCC. Considering that sustained cell growth is a distinctive hallmark of cancer cells, we performed a colony formation analysis to validate whether miR-145 stimulates ESCC cell growth by transfecting miR-145 or miR-145-in into Eca109 or TE-1 cells. The results showed that miR-145 mimic-transfected cells displayed fewer and smaller colonies than the controls (Figure 7A, upper panel). Conversely, TE-1 cells displayed higher number and larger colonies than the controls after transfection with miR-145 inhibitor (Figure 7B, upper panel, Figure 7C, left panel). Escaping apoptosis is another superior characteristic of tumor cells to obtain limitless growth. Analysis showed that the numbers of apoptotic cells significantly increased in miR-145-transfected Eca109 cells (Figure 7A, lower panel) but were decreased in miR-145-in-transfected TE-1 cells (Figure 7B, lower panel; Figure 7C, right panel). Western blot analysis further showed that the expression of apoptosis-related proteins such as Bax, cleaved PARP, caspase7, caspase3, and cleaved caspase3 was enhanced in miR-145 mimic-transfected Eca109 but decreased in miR-145-in-transfected TE-1 (Figure 7D). In addition, the c-Myc, a basic helixloophelix lencine zipper transcription factor capable of stimulating both cell proliferation and apoptosis, can stimulate cell proliferation and apoptosis and is previously confirmed as direct target gene for miR-145 [33]. We also found that the ectopic expression of miR-145 reduced the expression of c-Myc protein, whereas miR-145 depletion enhanced the protein expression levels (Figure 7D).

Transwell insert chambers were used to investigate the effect of miR-145 on the motility of esophageal cancer cells. Enhancing miR-145 expression in Eca109 cells by introducing miR-145 mimic effectively suppressed Eca109 cell migration, whereas transfection with miR-145 inhibitor increased the motility of TE-1 cells (Figure 7E and 7F). Considering that EMT facilitates cancer cell migration and leads to cancer metastasis, we tested whether miR-145 is involved in EMT and influences esophageal cancer metastasis. Figure 7G showed that miR-145 mimic enhanced E-cadherin expression and reduced vimentin expression in Eca109 cells, whereas miR-145 inhibitor yielded opposite results in TE-1 cells. Furthermore, cell motility is also regulated by fascin 1 (FSCN1), an actin-bundling protein and an integral component of invadopodia [34]. Studies showed that miR-145 inhibits the migration and invasion of nasopharyngeal carcinoma and lung cancer cell lines through FSCN1 downregulation [35, 36]. In the present study, overexpressing miR-145 reduced the expression of FSCN1 protein, whereas downregulating miR-145 increased FSCN1 protein expression levels in ESCC cell lines (Figure 7G).

MiR-145 inhibitor-treated ESCC cells lost their cell-to-cell adhesion and exhibited a spindle-like morphology, whereas control and miR-145 mimic-treated ESCC cells maintained a cobblestone-like epithelial appearance (Figure 7H). These findings indicate that miR-145 may affect cytoskeleton dynamics, as well as cell motility and migration. To test our hypothesis, we used F-actin to monitor the cytoskeleton dynamics of Eca109 cells. As shown in Figure 7H, Eca109 cell lines transfected with miR-145 mimic inhibited the formation of lamelipodia and filopodia compared with those transfected with miR-145 inhibitor. Hence, miR-145 may promote cancer cell motility and migration by controlling the cytoskeleton dynamics in esophageal cancer cells.

**Figure 8:**
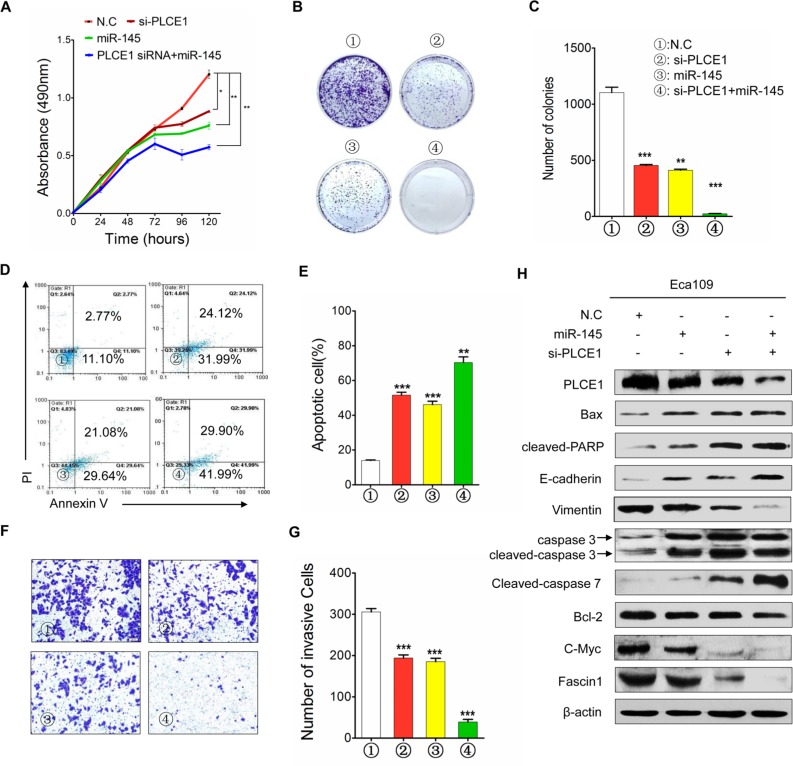
MiR-145 functions as tumor suppressor by inhibiting PLCE1 in ESCC (**A**) Cell proliferation in Eca109 cells at 24, 48, 72, 96, and 120 h after transfection. (**B**) Colony formation in Eca109 cells after transfection. (**C**) Histograms indicate the relative percentage of colony in the indicated clones. (**D**) Cell apoptosis detected by Annexin-V/propidium iodide combined with labeled flow cytometry in Eca109 cells 48 h after transfection. (**E**) Apoptotic evaluation was performed by obtaining the percentage of apoptotic cells. (**F**) Cell invasion in Eca109 cells after transfection. (**G**) Histograms indicate the relative percentage of cells across a Matrigel membrane with 8 mm pores. (**H**) Expression of PLCE1, apoptosis-related proteins, c-Myc, Fascin1, and EMT-related marker proteins in Eca109 cells co-transfected with miR-145 mimic and si-PLCE1 by using HiPerFect transfection reagents (Qiagen, Hilden, Germany) according to the manufacturer's instruction. *P < 0.05; **P < 0.01; ***P < 0.001 vs. scramble control (Student's *t*-test). ①: NC; ②: si-PLCE1; ③: miR-145; and ④: si-PLCE1 + miR-145.

We also examined whether PLCE1 knockdown by siRNA can potentiate the antitumor effects of miR-145. Co-transfection of si-PLCE1 and miR-145 mimic in Eca109 cells efficiently reduced PLCE1 expression and enhanced miR-145 expression, as well as partially increased miR-145-induced antitumor effects (Figure 8). These results suggest that miR-145 suppressed PLCE1 expression by binding directly to the 3′-UTR of PLCE1, and the negative regulation of PLCE1 by miR-145 may contribute partially to the antitumor effects of miR-145 in ESCC.

### PLCE1 is negatively correlated with miR-145 in ESCC tissues

**Figure 9:**
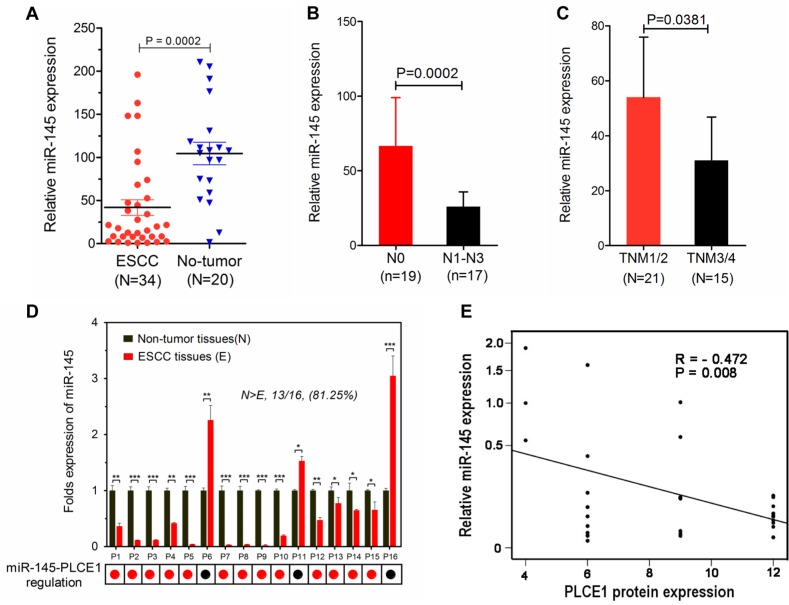
MiR-145 downregulation is inversely correlated with PLCE1 expression in human ESCC tissues (**A**) Expression levels of miR-145 in esophageal carcinoma tissues were measured by real-time PCR and quantified as described in the methods. The plot showed that miR-145 was significantly downregulated in ESCC compared with that in non-tumor tissues. (***P = 0.0002). (**B** and **C**) MiR-145 expression was negatively correlated with N classification and clinical staging. (**D**) Real-time PCR analysis of 16 paired ESCC tissues, where PLCE1 was significantly overexpressed as revealed by immunohistochemistry analysis (Figure 1A). MiR-145 was remarkably downregulated in all tested esophageal carcinoma tissues compared with that in the corresponding non-tumor cells. miR-145 expression was normalized to U6 (mean ± s.d., *n* = 3). *P < 0.05, **P < 0.01, ***P < 0.001 vs. non-tumor tissues (Student's *t*-test). (**E**) Inverse correlation of miR-145 expression with PLCE1 protein expression in human ESCC tissues (Pearson correlation analysis, R = −0.472, **P = 0.008). Data are presented as means of triplicate experiments.

MiRNAs have expression patterns that are opposite to those of their targets [37, 38]. To validate our findings, we applied real-time PCR to recapitulate miR-145 expression in a cohort of Kazakh patients with ESCC and presented the data in a scattered plot (Figure 9A). Considering that miR-145 was highly expressed in noncancerous tissues, we analyzed the correlations of its expression with clinical TNM classification. The expression levels of miR-145 were negatively correlated with TNM classification and clinical staging (Figure 9B and 9C). We also investigated whether PLCE1 protein expression is inversely correlated with miR-145 expression in a subset of human ESCC tissues. We performed real-time PCR analysis of 16 paired ESCC from the Han ethnic group. The results showed that PLCE1 was significantly overexpressed in ESCC, as evidenced by the immunohistochemistry analysis results (Figure 1A). Moreover, 13 of 16 (81.25%) esophageal cancer tissues expressed lower levels of miR-145 compared with the matched non-tumor tissues (Figure 9D). This finding indicates that the majority of ESCC tissues followed the expected miR-145-PLCE1 regulation pattern (as indicated by the red dots below the histogram); in this pattern, tumors showed lower levels of miR-145 and higher levels of PLCE1 compared with those in the matched non-tumor tissues. Furthermore, Pearson correlation showed a significant inverse correlation between miR-145 and PLCE1 protein expression in esophageal carcinoma tissues from the Chinese Han ethnic group (R = −0.472, **P = 0.008) (Figure 9E). These results demonstrate that miR-145 directly regulates PLCE1 and may function as a tumor suppressor by regulating the abnormal activity of PLCE1 in patients with ESCC from the Han and Kazakh ethnic groups.

## DISCUSSION

PLCE1, which differs from other molecules of the PLC family [39], is associated with cellular differentiation and apoptosis through its coaction with Ras family proteins [40]. Although PLCE1 has been extensively studied, its role in human cancer remains controversial. Previous findings demonstrated a significant reduction in PLCE1 expression in Ras-driven cancers, such as colorectal, lung, and skin tumors; as such, PLCE1 functions as a tumor-suppressor gene [41–43]. However, PLCE1 has also been identified as a novel candidate oncogene and a potential therapeutic target for several types of human cancers, such as bladder cancer [17, 44] and head and neck cancer [18]. Therefore, the function of PLCE1 may be tumor specific and depends on the stage of tumorigenesis.

Three-scale genome-wide association studies on the Chinese Han population revealed that PLCE1 is a susceptibility gene in ESCC [6–8]. Our previous study showed that PLCE1 may serve as a candidate marker for ESCC susceptibility in the Kazakh population; linkage disequilibrium variants may also influence ESCC risk individually and jointly by promoting mRNA and protein expression of PLCE1 [9]. Moreover, the heterozygote of PLCE1 rs2274223 increases susceptibility to HPV infection in Kazakh patients with esophageal carcinoma [13]. These findings suggest that PLCE1 may increase the risk for esophageal cancer because this gene affects epidemiologic and etiologic factors involved in ESCC carcinogenesis, which is, in turn, regulated by the genotype–phenotype of PLCE1. This finding contradicts those in previous reports on PLCE1 expression. We previously reported that PLCE1 protein expression is upregulated in Kazakh patients with ESCC [22]; we also confirmed that this protein functions as an oncogene and can induce inflammation and promote esophageal cancer formation through interaction with the NF-κB signal pathway [32]. These results are similar to those reported by Wang *et al.* [7] who studied the Chinese Han population, but differ from those stated by Hu *et al.* [23] who found that PLCE1 mRNA expression level is lower in ESCC than that in normal tissues; nevertheless, no significant difference was observed in the IHC score between ESCC and normal match. The abnormal PLCE1 expression and its possible carcinogenesis leading to dysregulation of PLCE1 in ESCC have not been completely elucidated. As such, in the present study, we comprehensively investigated the function of PLCE1 in esophageal cancer. We found that high PLCE1 protein levels in Han patients with ESCC are significantly correlated with poor patient survival. In addition, increased PLCE1 expression levels influence ESCC cell proliferation, as well as esophageal carcinoma cell migration and invasion. The present study provides the first evidence that miR-145 functions as a tumor suppressor by regulating aberrant PLCE1 activity in ESCC.

This study is the first to report that PLCE1 protein expression increased progressively from normal esophageal epithelium to low-grade intraepithelial neoplasia to ESCC and reached the highest expression level in the high-grade intraepithelial neoplasia in the Han ethnic group. High PLCE1 expression is correlated with poor prognosis of ESCC patients, suggesting that PLCE1 is a potential biomarker for ESCC diagnosis and treatment. The present findings are consistent with those of Wang *et al.* [7] and previous meta-analysis on PLCE1 protein expression in upper gastrointestinal cancer [45], as well as in other human malignancies, such as gastric [14] and bladder cancer [17]. However, the elevated PLCE1 protein expression patterns in ESCC differ from those reported by Hu *et al.* [23], who found no difference in PLCE1 IHC scores between ESCC and the matching adjacent normal tissues. Thus far, the precise and detailed biological significance of PLCE1 overexpression in ESCC remains poorly understood.

PLCE1 acts as an effector of GTPases and therefore influences cell growth, differentiation, apoptosis, and angiogenesis [46]. In the present study, PLCE1 knockdown reduced cell growth/proliferation and increased the frequency of apoptotic esophageal cancer cells. Similarly, PLCE1 knockdown in bladder cancer reduces proliferating cell nuclear antigen and cyclin D1 in the bladder tumor xenograft, leading to significant inhibition of cell proliferation and cell cycle arrest [47]. Apoptosis-related proteins were also assessed using Western blot analysis. Total p53 was markedly upregulated in PLCE1-silenced ESCC cells compared with that in the control group, similar to those reported by Yun Li *et al.* [48]. We further found that the inhibited expression of PLCE1 significantly enhanced cleaved PARP and cleaved caspase3 expression, which is a crucial executioner of cell apoptosis, but disrupted the balance of the Bcl-2 family members by reducing Bcl-2 expression and increasing Bax expression. Moreover, p53 plays an important role in apoptosis via the mitochondrial dependent pathway. Bcl-2 family proteins, which are the downstream mediators of p53-dependent apoptosis, are key regulators in the apoptotic pathway. These results indicate that PLCE1 silencing partially activated apoptosis by regulating the p53-Bax/Bcl-2-caspase3 mediated pro-apoptotic signaling pathway. This study also reveals that PLCE1 knockdown enhanced cell apoptosis induced by TNFα, TRAIL, PTX, and 5-FU, suggesting that overexpressing PLCE1 contributed to the resistance of ESCC cells to chemotherapy. Therefore, PLCE1 is an effective candidate biomarker and a potential therapeutic target in ESCC because it can restore cell sensitivity to apoptosis or induce apoptosis to eliminate cancer cells.

PLCE1 encodes a novel Ras-related protein (R-Ras) effector and mediates the effects of R-Ras on actin cytoskeleton and membrane protrusion. Previous reports suggested that PLCE1 is co-precipitated with R-Ras protein and enhances cell membrane protrusion and migration [49, 50]. PLCE1 silencing may also downregulate MMP and BCL2 gene expression, thereby reducing the invasiveness of bladder cancer cells [17]. Similarly, the present findings confirmed that PLCE1 controlled cytoskeleton dynamics by increasing F-actin expression, an indicator of cytoskeleton dynamics, and by promoting the EMT of esophageal cancer cells, thereby altering esophageal cellular shape, motility, and migration. In addition, signaling pathways controlling cytoskeletal dynamics are altered during malignant transformation. Increase in cell motility allows cancer cells to invade surrounding tissues and metastasize. Bourguignon *et al.* [46] demonstrated that HA activation of RhoA-PLCE1 stimulates intracellular Ca^2+^ mobilization and Ca^2+^/calmodulin-dependent kinase II, leading to phosphorylation of the cytoskeletal protein and filamin and promotion of cancer cell migration in human head and neck squamous carcinoma. These reports support our assumption that oncogenic PLCE1 is involved in esophageal metastasis. Thus, PLCE1 is likely to become a focus of esophageal cancer treatment. However, regulation of PLCE1 activity or expression leading to oncogenic PLCE1 overexpression has not been studied.

PLCE1 overexpression in esophageal cancer cells is caused by multiple factors, including genetic variants of PLCE1, as shown in our previous report [9], and miRNA regulation, as revealed in the present study. MiRNAs generally target protein-coding genes *in vivo* by binding to the characteristic binding sites in the 3′-UTRs of their targets. The present study provides the first evidence that miR-145 inhibits proliferation and invasion of esophageal cancer by binding directly to the 3′-UTR of PLCE1. Our previous research and that of Wang *et al.* [51] showed that the PLCE1 variant rs2274223 enhances PLCE1 mRNA and protein expression in esophageal cancer tissues and ESCC cell lines. However, the mechanisms contributing to PLCE1 overexpression in esophageal cancers have not been reported yet. To our knowledge, few lines of evidence support that miR-145 may regulate PLCE1 function in cancer.

MiR-145 is one of the most downregulated miRNAs in a number of human cancers. Khan *et al.* [52] reported that miR-145 is significantly downregulated in human pancreatic tumor compared with that in non-tumor pancreatic tissues. Wang *et al.* [53] also reported that miR-145 is downregulated in NSCLC specimens, significantly correlated with advanced clinical stage and lymph node metastasis, and functions as a tumor suppressor. Similar to the report of Wang *et al.* [36], the present study confirmed that miR-145 expression was reduced in ESCC cell lines and tissues from the Han ethnic group. In particular, the reduced miR-145 expression was significantly correlated with lymphatic metastasis and TNM staging in patients with ESCC. Nevertheless, limited information is known about the underlying cause of miR-145 downregulation in esophageal cancer. Suh *et al.* found that miR-145 downregulation is mediated by DNA methylation and p53 mutation pathways [54]. Sachdeva *et al.* suggested that a regulatory system of miR-145 involving the Akt and CCAAT/enhancer binding protein beta may downregulate miR-145 in cancer cells [55]. MiR-145, which is located in chromosome 5q32–33, exhibits frequent deletion in esophageal cancer, as detected through comparative genomic hybridization [56], and may harbor many tumor suppressor genes [57]. MiR-145 also exhibits anti-tumorigenic activity and is involved in various cancer-related events, such as cancer cell proliferation, invasion, and migration [58]. The present study showed that miR-145 overexpression in ESCC reduced cell proliferation and clonogenicity but increased apoptosis by elevating the expression levels of Bax, cleaved PARP, cleaved caspase3, and C-Myc. Furthermore, miR-145 overexpression consistently reduced ESCC migration by controlling the cytoskeletal dynamics and by regulating the EMT of the ESCC cell lines. Cytoskeletal dynamics are also altered during malignant transformation. Increased cell motility allows cancer cells to invade surrounding tissues and metastasize. Similar to those reported by several studies [36, 59], the present findings suggest that miR-145 acts as tumor-suppressive miRNA in ESCC. Moreover, miR-145 modulated the oncogene PLCE1, which may explain why miR-145 downregulation during esophageal carcinogenesis can promote cancer progression. Therefore, an effective drug delivery system involving miR-145 for esophageal cancer therapy must be developed in future studies.

Several miR-145 targets can mediate the tumor-suppressor function of miR-145 in several cancer types [60–62]. However, targeting PLCE1 may be one mechanism through which miR-145 exerts its tumor-suppressive function in esophageal cancer. In the present study, the bioinformatics analysis indicated that miR-145 possibly targets hundreds of mRNAs. We experimentally validated several of the predicted genes, including PPP3CA, CBFB, YES, and STAT1 [63, 64]. MiR-145 targets the SOX9/ADAM17 axis in the head and neck squamous cell carcinoma [65]. A recent study has reported two new miR-145 targets, namely, the metastasis gene FSCN1, which is regulated in nasopharyngeal carcinoma cell line invasion and metastasis [35] and c-Myc, and TPD52, which is regulated in brain metastasis of lung cancer [33]. The present study showed that miR-145 involved in ESCC invasion and migration *in vitro* via transduction of miR-145 or miR-145 inhibitor into Eca109 and TE-1 cells significantly suppressed not only the expression of PLCE1 but also that of c-Myc and FSCN1; the two latter genes are previously identified targets of miR-145. Moreover, *in vitro* 3′-UTR luciferase assay analysis confirmed that miR-145 exerted its effects by targeting the 3′-UTR of PLCE1. MiR-145-mediated PLCE1 downregulation also suppressed ESCC aggressiveness in terms of cell growth, proliferation, metastasis, and invasion. Co-transfection of PLCE1 siRNA with miR-145 mimic into the Eca109 esophageal cancer cells revealed that the transfection attenuated the elevated PLCE1 expression. These findings show that miRNA-processing machinery is required for miR-145 to function as endogenous regulator of oncogenic PLCE1 in esophageal cancer. Furthermore, miR-145 and PLCE1 expression in human ESCC clinical specimens were found to be significantly inversely correlated. Each miRNA contains approximately 100 target sites and regulates the expression of hundreds of mRNAs. In the present study, miR-145 may act as tumor suppressor at least partially by inhibiting PLCE1 hyperactivity in ESCC; this finding demonstrates a novel mechanism of PLCE1 overexpression in ESCC tissues.

In conclusion, high PLCE1 expression levels are correlated with poor prognosis of patients with ESCC, and this molecule functions as an oncogene in ESCC tumorigenesis. We comprehensively demonstrated the suppressive role of miR-145 on PLCE1 expression and esophageal cancer cell proliferation and invasion. PLCE1 expression levels serve as potential biomarkers of ESCC, and delivery of PLCE1-targeting miR-145 is a candidate therapeutic approach for preventing tumor proliferation and metastasis of esophageal cancer.

## MATERIALS AND METHODS

### Patients and tissue specimens

This study included 112 Han patients with ESCC treated in the First University Hospital, Shihezi University School of Medicine between 2009 and 2011. Tissue microarrays (TMAs) were used in immunostaining PLCE1. All tissue samples were collected from patients who underwent pathological examination and esophagectomy without prior chemotherapy or radiotherapy. No restrictions were set in terms of age, sex, or disease stage. Clinical–pathological data from tumor differentiation and lymph node metastasis were also collected. All cases with pathologic diagnoses for tumor-node-metastasis (TNM) stages were evaluated according to the seventh edition of Cancer Stage Manual issued by the American Joint Committee on Cancer in 2009. Among 112 ESCC specimens, 99 specimens that matched the adjacent normal esophageal tissues were used as controls. Approximately 99 precursor lesions in the adjacent mucosa were also selected; 60 of which were classified as low-grade intraepithelial neoplasia (LGIN) and 40 as high-grade intraepithelial neoplasia (HGIN). All cases were diagnosed by two pathologists. Additional clinical–pathological data of all patients were obtained from their medical records and are listed in Supplementary Table 3. Follow-ups were conducted on 75 Han patients and ended on 10 July 2014. The clinical–pathological characteristics of the 75 patients with follow-up information are also presented in Supplementary Table 3. The research protocol employed in this study was approved by the Institutional Review Board of Shihezi University School of Medicine.

### PLCE1 expression detected by immunohistochemistry (IHC) using TMAs

Paraffin-embedded materials were sampled from 112 formalin-fixed esophageal cancer tissues, 99 precursor lesion tissues, and 99 normal tissue samples with 0.6 mm-diameter tissue cores using a tissue arrayer (ALPHELYS, Plaisir, France). Tumor samples were fixed with 10% formalin in PBS, The paraffin-embedded 4 μm sections were baked at 65°C for 60 min, and then rehydrated using graded alcohols. Each 4 μm tissue section was deparaffinized and rehydrated. The sections were autoclaved in ethylenediaminetetraacetic acid buffer (pH 9.0) at 130°C for 10 min in a microwave oven, cooled to 30°C for 40 min, and incubated with fresh 3% H_2_O_2_ in methanol for 10 min at room temperature. Tissue sections were then incubated at 4°C overnight with anti-PLCE1 (HPA015598; Sigma-Aldrich Co., St. Louis, MO, USA; 1:50 dilutions). Negative controls were prepared by replacing the primary antibodies with PBS. The tissues were washed three times in PBS for 5 min, and then incubated with secondary antibody for 30 min at 37°C. Subsequently, 3,3-diaminobenzidine was employed to visualize PLCE1 antibody binding; the tissue sections were counterstained with hematoxylin.

### Semi-quantitiative assessment and scoring

PLCE1 expression was scored semi-quantitatively according to the percentage of positive cells and cytoplasmic/nuclear staining intensity. The results were assessed by two investigators. The percentage of positively stained cells was as follows: 0 (< 5% positive cells), 1 (6%–25% positive cells), 2 (26%–50% positive cells), 3 (51%–75% positive cells), or 4 (> 75% positive cells). The cytoplasmic/nuclear staining intensity was categorized as follows: 0 score, negative; 1 score, buff; 2 score, yellow; and 3 score, brown. The percentage of positive epithelial cells and staining intensities were then multiplied to obtain the immunoreactivity score (IS) for each case. For example, if the staining intensity was brown (3) and the percentage of positive cells was greater than 45% (2), the IS would be 3 × 2 = 6. Two pathologists independently reviewed five random fields from each sample slide. Cases with discrepant scores were reviewed using a 10-headed microscope and reassigned a consensus score. Thus, the IS ranged from 0 to 12. Optimal cut-off values for this assessment system were identified as follows: high expression of PLCE1 was defined as an expression index score of 5, while low expression as an expression index score of < 5. These cases were divided into two groups based on their PLCE1 staining IS. Cases with a score of ≥ 5 were categorized as the high expression group, while those with a score of < 5 were categorized as the low expression group.

### Cell culture and transfections

Five esophageal cancer cell lines (Eca109, EC9706, TE-1, KYSE-150, and KYSE-450) were purchased from the Institute of Biochemistry and Cell Biology of the Chinese Academy of Sciences (Shanghai, China). Cells were maintained in Dulbecco's modified Eagle's medium (DMEM) or RPMI 1640 (Gibco) supplemented with 10% fetal bovine serum (Gibco), 100 units of penicillin/ml (Sigma) and 100 mg of streptomycin/ml (Sigma) in humidified air at 37°C with 5% CO_2_. The miRNA-145-mimics (miRNA-145-mimic), antisense miRNA-145-inhibitor, siRNA against human PLCE1, the relative negative scramble control RNA oligos with no significant similarity to human genome DNA by BLAST search (Scramble) were synthesized by Qiagen (Hilden, Germany). RNA transfections were performed at a final concentration of 50 nM, using HiPerFect transfection reagents (Qiagen, Hilden, Germany) in serum-free conditions following the Quick-Start Protocol. The target sequences for PLCE1-siRNA1 and PLCE1-siRNA2 are 5′-AGC GUU GGU CCA UGC UUA ATT-3′ and 5′-GGG UCU UGC CAG UCG ACU ATT-3′, respectively.

### RNA isolation and quantitative real-time PCR

Total RNA was extracted from the ESCC tissues and ESCC cell lines using the miRNA or mRNA Extraction Kit (Qiagen, Hilden, Germany) according to the protocol of the manufacturer. The cDNA of miRNA or mRNA were synthesized with One Step PrimeScript miRNA or mRNA cDNA Synthesis Kit (Qiagen). Quantitative real-time PCR (qPCR) was performed using the SYBR green Premix Ex Taq II (Qiagen) with StepOne Plus Real-Time PCR System (Applied Biosystems). The expression of U6 was used as endogenous control for detection of miRNA expression level. The expression of β-actin was used as endogenous control for detection of mRNA expression level. The reaction was performed on an ABI Prism 7500 Sequence Detection System (Applied Biosystems, Foster City, CA, USA). The 2^−ΔΔCt^ method was used to quantify the expression of miR-145 and PLCE1.

### Western blot analysis

Transfected cells were lysed in RIPA lysis buffer (Solarbio). Protein was loaded onto a 10% SDS-polyacrylamide gel electrophoresis, which was then transferred to PVDF membranes (Immobilon 0.45 μm, Millipore, USA), and immersed in a blocking solution containing 5% non-fat milk and 0.1% Tween-20 for 1 h. After blocking, membranes were incubated with PLCE1, E-cadherin (Santa Cruz Biotechnology, Santa Cruz, CA), Bax, cleaved-PARP, vimentin, P53, Snail, Slug, Fascin1 (Abcam, MA, USA), C-Myc, caspase 3 (Proteintech Group, Inc., Chicago, IL, USA), Bcl-2 (Beyotime, China) at 4°C overnight, and then with secondary antibodies for 2 h at room temperature. After washing, the resulting bands were visualized using the standard ECL procedure, quantified by densitometry, and normalized to the corresponding β-actin bands.

### Immunofluorescence analysis

Eca109 cells were transfected with si-PLCE1 or miR-145-mimic and miR-145-inhibitor. At 48 h, the cells were seeded onto fibronectin-precoated coverslips (BD BioCoat; BD Biosciences). After an overnight incubation, the cells were washed twice with cold PBS, fixed with 4% formaldehyde for 20 min at room temperature, and permeabilized with 1% Triton X-100 for 4 minutes. The cells were incubated with anti-PLCE1 (Sigma; 1:50 dilution) and anti-F-actin (Abcom; 1:10 dilution) in 5% bovine serum albumin at 4°C overnight. Afterward, the primary antibody was discarded, and the cells were washed three times with PBS and incubated with the appropriate second antibody for 30 min. After washing with PBS, the coverslips were mounted with mounting medium (Vectashield; Vector Laboratories). Cells were then observed and photographed under a confocal microscope (Zeiss LSM510, Carl Zeiss).

### Luciferase reporter assay

ESCC cells were maintained in DMEM; cells at 70–90% confluence in 96-well plates were allowed to settle for 16 h, and then co-transfected with 200 ng of pMIR vectors (Promega), which harbored PLCE1 3′-UTR wild-type or mutant constructs and 10 ng of the Renilla luciferase vector with Lipofectamine^®^ 2000 Reagent (Invitrogen) according to the recommendation of the manufacturer. Luciferase and Renilla signals were measured at 48 h after transfection using the Dual Luciferase Reporter Assay Kit (Promega) according to a protocol provided by the manufacturer. Three independent experiments were performed and the data were presented as the mean ± SD.

### 3-(4, 5-Dimethyl-2-thiazolyl)-2, 5-diphenyl-2H-tetrazolium bromide (MTT) assay

Cell proliferation was analyzed using MTT assay. Cells (4 × 10^3^) were seeded on 96-well flat-bottom plates (NUNC). After culturing for 24, 48, 72, 96, and 120 h, the cells were stained with 20 μL of sterile MTT dye (5 mg/mL, Solarbio) for 4 h at 37°C, followed by removal of the culture medium and addition of 150 μL of dimethyl sulfoxide. The 96-well plates were then shaken until the formazan crystals dissolved completely. The absorbance value was measured on a microplate reader (Bio-Rad) at 490 nm. All experiments were performed in triplicate.

### Cell apoptosis assay

Cells were transfected with si-PLCE1 or miR-145-mimic and miR-145-inhibitor, and then cultured in 24-well plates. At 48 h after transfection, cells were removed from the plate using a trypsin digestion solution, collected, and resuspended in 500 μL of 1x binding buffer. After the addition of 5 μL of Annexin V-FITC to each well, cells were incubated in the dark for 5 min. An aliquot of 10 μL of PI was added to each well, followed by additional incubation in the dark for 5 min. Finally, flow cytometry was performed.

### Colony formation assay

Cells were transfected with si-PLCE1 or miR-145-mimic and miR-145-inhibitor, 3 × 10^3^ cells were seeded on each six-well plates. After 14 days, the cells were washed with 1 mL of PBS, fixed with 4% paraformaldehyde for 15 min, stained with 0.1% crystal violet for 20 min, and finally washed three times with 1 mL of water. The number of colonies was manually counted and differences were assessed by two-tailed *t*-test. The experiment was performed independently three times for each cell line.

### Cell migration and invasion assays

Cells were infected with si-PLCE1 or miR-145-mimic and miR-145-inhibitor for 48 h, and then seeded onto a synthetic basement membrane in the inset of a 24-well culture plate. A 24-well transwell plate (Corning) was used to measure the migratory and invasive abilities of each cell line. For the invasion assay, polycarbonate filters coated with 40 μL of Matrigel (1:8, BD Bioscience) were placed in a Transwell chamber, and then 5 × 10^4^ cells were plated in the top chamber. For the migration assay, 2.5 × 10^4^ cells were plated in the top chamber of the transwell plate, which was lined with a non-coated membrane. In both assays, the top chamber was loaded with 200 μL cell suspension and the lower chamber was filled with 600 μL of DMEM with 20% fetal calf serum as a chemoattractant. After incubation at 37°C in 5% CO_2_ for 24 h, filters were fixed with 4% paraformaldehyde for 15 min and stained with 0.1% crystal violet for 20 min. Non-invading cells were removed using a cotton swab and invading cells on the underside of the filter were counted with an inverted microscope. The mean of triplicate assays for each experimental condition was calculated.

### Statistical analysis

The SPSS software V17.0 was used in statistical analysis. All experiments were performed at least three times, and all samples were analyzed in triplicate. Results are presented as mean ± standard deviation. Student's *t*-test and two-way ANOVA were used to determine significance differences. Chi-square test was used to compare the expression of PLCE1 by IHC among different groups. Moreover, correlations between prognostic outcomes and PLCE1 expression were investigated using Kaplan–Meier analysis and Cox proportional hazards model. Univariable and multivariable Cox proportional hazard regression models were also applied to identify factors independently associated with overall survival. *P* < 0.05 was considered statistically significant.

## SUPPLEMENTARY MATERIAL TABLES


