# Supplementary material for: Targeting oncogenic PLCE1 by miR-145 impairs tumor proliferation and metastasis of esophageal squamous cell carcinoma
Source: Oncotarget. 2015 Dec 8;7(2):1777–95. doi: 10.18632/oncotarget.6499 (PMC4811497; doi:10.18632/oncotarget.6499)
Supplement: Supplementary file 1 [file oncotarget-07-1777-s001.pdf]

Targeting oncogenic PLCE1 by miR-145 impairs tumor proliferation and metastasis of esophageal squamous cell carcinoma

Supplementary Materials

Supplementary Table S1: PLCE1 protein expression during cancer progression by IHC analysis in Chinese Han population

Cancer progression	Immunostaining		*P* value
	Low	High	
normal esophageal epithelium①	97 (97.97)	2 (2.03)	①:② $P < 0.001$; ①:③ $P < 0.001$
Low grade intraepithelial neoplasia②	25 (41.67)	35 (58.33)	②:③ $P = 0.174$; ②:④ $P = 0.046$
high grade intraepithelial neoplasia③	11 (27.50)	28 (72.50)	③:④ $P = 0.864$
ESCC④	30 (26.78)	82 (73.22)	①:④ $P < 0.001$

Supplementary Table S2: Univariate and multivariate analyses of overall survival in esophageal carcinoma

Variables	Univariate analysis			Multivariate analysis		
	HR	95% CI	*P* value	HR	95% CI	*P* value
Gender (male vs. Female)	1.409	0.698–2.844	0.339	1.049	0.492–2.234	0.902
Age ≥ 50 vs.< 50 years	1.014	0.975–1.055	0.497	0.999	0.958–1.042	0.969
Differentiation G2–G3 vs. G1	0.772	0.384–1.553	0.469	1.298	0.605–2.784	0.503
T classification T3–T4 vs. T1–T2	1.092	0.549–2.173	0.802	1.119	0.486–2.578	0.791
Lymph node metastasis N2–N3 vs. N0–N1	1.344	0.665–2.715	0.410	0.546	0.147–2.032	0.367
Clinical stage III–IV vs. I–II	1.722	0.828–3.580	0.146	1.130	0.299–4.275	0.857
PLCE1 expression High vs. Low	7.978	1.899–33.515	0.005	8.435	1.875–37.983	0.005

Note: HR: hazard radio, CI: confidence interval.

Supplementary Table S3: Demographic and Clinical Characteristics of ESCC Patients

	Total ESCC patients from Han ethnic (*N* = 112)		ESCC patients with follow-up information (*N* = 75)	
Characteristic	No.	%	No.	%
Age at surgery, years				
Median	63		65	
Range	34–83		42–73	
Sex				
Male	81	72.32	48	64.00
Female	31	27.68	27	36.00
Differentiation				
Well	34	30.35	26	34.67
Moderate	63	56.25	36	48.00
Poor	15	13.40	13	17.33
Lymph node metastasis				
No	69	61.61	50	66.67
Yes	43	38.39	25	33.33
TNM				
I + II	79	70.53	53	70.67
III + IV	33	29.47	22	29.33
